# Historical Highlight: The Chemical Characterization of the Pneumococcal Transforming Principle

**DOI:** 10.20411/pai.v8i2.687

**Published:** 2024-02-28

**Authors:** Neil S. Greenspan

**Affiliations:** 1 Case Western Reserve University, Cleveland, Ohio

**Keywords:** DNA, gene, phenotype, capsular polysaccharide, virulence

The editors of *Pathogens and Immunity* are commemorating this month, the 80^th^ anniversary of the publication of the landmark article by Oswald Avery, Colin MacLeod, and Maclyn McCarty in the *Journal of Experimental Medicine* on February 1, 1944 [1]. The study by Avery et al determined with extraordinary rigor the chemical nature of the so-called transforming principle inferred to exist by Frederick Griffith based on experiments he published in 1928. This entity, derived from heat-killed encapsulated and virulent type III *Streptococcus pneumoniae*, was so-named because it possessed the ability to transform, in mice or *in vitro*, live non-encapsulated type II *Streptococcus pneumoniae* lacking virulence into live and virulent type III pneumococci. Avery and his colleagues showed that the transforming principle was composed of DNA, not protein, polysaccharide, or lipid.

In other words, Avery et al provided the first concrete evidence supporting the now routine notion that genes in cells, bacterial cells in this instance, are made of DNA. An alternative way to express the same claim is that the study by Avery and colleagues was the beginning of a long series of studies that established that DNA provides the material substrate for biological inheritance in cells and organisms.

Their 1944 study is also notable for additional reasons.

- It arguably provided, along with Griffith's prior work, the first understanding of horizontal gene transfer among microorganisms.
- It demonstrated a connection between genetic variation and virulence.
- It is a marvelous example of using multiple independent experimental methods to build a case for a critical conclusion.

Therefore, we commend to biomedical science students and their mentors the study of this study. We might anticipate that the article itself will represent a higher-order transforming principle in its own right that operates in the domain of cognition as opposed to genetics.
