# Erratum to: “The Chemical Characterization of the Pneumococcal Transforming Principle. Pathogens and Immunity. 2024;8(2):177–178. doi: 10.20411/pai.v8i2.687”

**DOI:** 10.20411/pai.v9i1.704

**Published:** 2024-04-02

**Authors:** Neil S. Greenspan

**Affiliations:** 1 Case Western Reserve University, Cleveland, Ohio

**Keywords:** DNA, gene, phenotype, capsular polysaccharide, virulence

## Erratum

In the original publication, “The Chemical Characterization of the Pneumococcal Transforming Principle. Pathogens and Immunity. 2024;8(2):177–178. doi: 10.20411/pai.v8i2.687,” the title did not clearly distinguish this commentary from the originally published study.

The title and the section header were changed to more clearly denote this article as a commentary on the originally published study.

